# Impact of Immediate and Delayed Breast Reconstruction on Quality of Life of Breast Cancer Patients

**DOI:** 10.3390/ijerph19148546

**Published:** 2022-07-13

**Authors:** Stana Pačarić, Želimir Orkić, Marko Babić, Nikolina Farčić, Andrea Milostić-Srb, Robert Lovrić, Ivana Barać, Štefica Mikšić, Jasenka Vujanić, Tajana Turk, Zvjezdana Gvozdanović, Dragica Pavlović, Nika Srb, Ivana Pačarić

**Affiliations:** 1 Department of Surgery, University Hospital Centre Osijek, 31 000 Osijek, Croatia; marko.babic@kbco.hr (M.B.); nikfarcic@gmail.com (N.F.); turk.tajana@gmail.com (T.T.); 2 Faculty of Medicine Osijek, Josip Juraj Strossmayer University of Osijek, 31 000 Osijek, Croatia; zg9598@gmail.com (Z.G.); dragica.pavlovic@dzobz.hr (D.P.); srbnika@gmail.com (N.S.); 3 Nursing Institute “Professor Radivoje Radić”, Faculty of Dental Medicine and Health Osijek, Josip Juraj Strossmayer University of Osijek, 31 000 Osijek, Croatia; rlovric@fdmz.hr (R.L.); ivbarac@yahoo.com (I.B.); steficamiksic@gmail.com (Š.M.); jasenkav@gmail.com (J.V.); ipacaric1709@gmail.com (I.P.)

**Keywords:** breast cancer, mastectomy, breast reconstruction, quality of life

## Abstract

A mastectomy affects the psychological, social, and sexual well-being of patients. Research has confirmed that breast reconstruction is important for improving the quality of life in patients with breast cancer. The aim of this study was to assess the quality of life of patients who underwent a mastectomy followed by immediate or delayed breast reconstruction. This prospective study was conducted from January 2018 to March 2020 at the Clinical Hospital Center Osijek, using the health questionnaire SF-36. The study included 79 patients. The results of the study showed that patients who underwent a mastectomy had the lowest scores in the domain of restriction due to physical difficulties, 18.8 (6.3–31.3), in physical functioning and limitation due to emotional difficulties, 16.7 (8.3–33.3), in mental health. In immediate breast reconstruction, patients rated better physical health (*p* < 0.001), while patients who underwent delayed breast reconstruction rated their mental health worse (*p* < 0.001) as measured by the SF-36 questionnaire. Conclusion: The results of this study show that patients without breast reconstruction rated their quality of life worse than patients who underwent immediate and delayed breast reconstruction after mastectomy. There is no difference in the quality of life between patients who underwent immediate and delayed breast reconstruction after mastectomy.

## 1. Introduction

Breast cancer is the most commonly diagnosed malignancy and the leading cause of death in women worldwide. According to the statistics, breast cancer is responsible for 30% of newly diagnosed cancer cases in women, and it is likely that one in eight women will develop breast cancer during their lifetime, while 14% of cases are associated with breast cancer mortality in women [1,2].

In the Republic of Croatia, breast cancer is a significant public health problem and is the second leading cause of death in women. According to the latest data from the Cancer Registry in Croatia, 2767 newly diagnosed women were registered in 2017, and 752 women died of this malignant disease in 2019 [3]. Diagnosis and treatment of breast cancer has an impact on a woman’s physical and emotional functioning as well as poor self-perception of body image. These problems are more evident during the early period after a mastectomy when women often feel tense and emotionally irritable [4].

Women with breast cancer may suffer from treatment-related side effects, such as scarring after mastectomy and lymphedema, and side effects from oncology therapy. Research has shown that these effects will lead to altered body image, problems with sexual dysfunction / intimacy as well as low self-esteem [5,6].

Breast reconstruction plays a significant role in the treatment of breast cancer. Breast removal affects the psychological, social, and sexual well-being of patients, including the need to discuss breast reconstruction after mastectomy, which has been popular in the treatment of breast cancer in the last decade [7,8]. Recently, the total number of breast reconstructions has increased significantly. Nowadays, breast reconstruction should be individualized, primarily taking into account not only the oncological aspects of the tumor, neo-/adjuvant treatment, and genetic predisposition, but also its time (immediate or delayed breast reconstruction), as well as the patient’s condition and desire [9].

Options for breast reconstruction mainly include the placement of breast implants or the use of the patient’s own tissue (autologous reconstruction). Breast reconstruction can be performed with several techniques, such as implantation of tissue expanders, breast reconstruction with implants and autologous tissue, and breast reconstruction with autologous tissue flaps. The flaps used in breast reconstruction are bound and free transverse rectus abdominis myocutaneous (TRAM) flap, then deep inferior epigastric perforator (DIEP) flap or superficial inferior epigastric artery (SIEA) free flap which are mainly used in secondary breast reconstruction. Saline-filled implants and silicone gel implants are safe and effective implant-based reconstruction options. Autologous reconstruction usually involves the transfer of abdominal tissue with recent advances that allow for the preservation of abdominal muscles. Implant reconstruction and autologous reconstruction have advantages and disadvantages and both types of reconstruction can be compromised by subsequent radiation therapy. For this and other reasons, consultation with a plastic surgeon at an early stage of treatment planning is important for women considering reconstruction after a mastectomy [10,11,12].

Patients undergoing a mastectomy have two options for breast reconstruction: immediate breast reconstruction (IBR) and delayed breast reconstruction (DBR). Research has shown that IBR has better outcomes than DBR in reducing the total number of surgical procedures and the associated risks. Additional benefits of immediate breast reconstruction include psychological well-being, reduced recovery time, better quality of life, and lower overall costs [13,14,15]. Immediate breast reconstruction as a single procedure with a standard implant is suitable for patients who after a mastectomy remain with the appropriate amount of skin and after a mastectomy that preserves skin or nipple. Mastectomy that preserves skin followed by immediate reconstruction gives the best aesthetic outcomes [16,17]. Delayed reconstruction may still be recommended for patients with significant medical comorbidities, planned radiation therapy (PMRT) after a mastectomy, inflammatory breast cancer, or in patients who are emotionally unwilling to make well-informed decisions about immediate breast reconstruction. Psychological and emotional stress that accompanies the diagnosis of breast cancer can compromise the patient’s ability to make decisions in that environment [18].

Over the last 30 years, significant technical advances in breast reconstruction have increased the effectiveness of this surgical technique as a means of potentially improving quality of life associated with health (HRQoL) for breast cancer survivors. Breast reconstruction studies are increasingly aimed at assessing outcomes based on the patient’s own perception of the surgical outcome and its effect on HRQoL, which is a multidimensional construct that examines three key domains: physical, mental and social domains [19,20]. The results of studies examining the quality of life showed that the quality of life was better in patients who underwent breast reconstruction compared to mastectomy without reconstruction. Patients with breast reconstruction were satisfied with the appearance of the breasts, physically and psychosocially they felt better with less pain and more sexually attractive [21,22].

Patients should be aware that mastectomy followed with breast reconstruction is a more complex operation and that complications can occur with any reconstruction. Patient expectations should be assessed prior to surgery to optimize satisfaction. Complications can occur with any type of breast surgery. The most common complications associated with breast reconstruction include seroma formation, infections, scars, hematomas, chronic back pain, lobe failure, abdominal weakness, bulge or hernia, and necrosis [23].

In the Republic of Croatia, a program for early detection of breast cancer is being implemented at the national level, and all women have a right to breast reconstruction after mastectomy financed by the state health insurance. However, in Croatia, a significant number of patients still do not decide on reconstruction after mastectomy. The reasons are different: lack of desire, fear of the procedure and possible problems or complications, lack of information, etc. Women who decide to subsequently reconstruct their breasts can contact plastic surgery in order to agree on the secondary reconstruction. A literature review on breast reconstruction conducted by Platt et al. demonstrated large variations across different countries and regions: 9.9% in Australia, 14% (immediate reconstruction: 1%, delayed reconstruction: 13%) in Denmark, 16.5% in England, and up to 42% in a network of tertiary care centers in the United States [24]. In Croatia, 43–50% of women would agree to breast reconstruction if recommended by the surgeon [25].

Research conducted in Croatia has shown that breast reconstruction has become increasingly popular in the last decade and is becoming a standard of health care for patients with breast cancer [26,27]. There have been few studies in Croatia that have assessed the quality of life of women after breast reconstruction [26,27].

The aim of this study was to investigate and compare the quality of life of patients who underwent a mastectomy, immediate and delayed implant-based breast reconstruction, with the SF-36 questionnaire which assessed the physical and mental components of the questionnaire.

## 2. Materials and Methods

This prospective study was conducted between January 2018 and March 2020 at the Department of Plastic Surgery, University Hospital Osijek after the approval of the Ethics Committee (R1-1574-4/2018) and was conducted in accordance with the Declaration of Helsinki. All participants were informed and agreed on the purpose of the research and the anonymity of the data, and that participation in the research was voluntary. The research was conducted with the Croatian version of the health questionnaire (Short form health survey—SF-36), clinical variables and sociodemographic questionnaire. In addition to the questionnaires, participants received a written explanation of the survey and written instructions on how to complete the questionnaire. All participants were asked for written consent to participate in the research, which was signed by the participants.

In the study, 100 patients were recruited, 15 patients were excluded from the study due to recurrent disease, and 85 patients remained in the study and filled out questionnaires after the end of treatment. After reviewing the questionnaires, 6 questionnaires were found to be incorrectly filled out and excluded from the survey.

The study included 79 patients between the ages of 34 and 68 who were diagnosed with breast cancer and underwent mastectomy and breast reconstruction at the Department of Plastic Surgery between the years 2018 and 2020. During breast reconstruction in our hospital, mastectomy is performed by plastic surgeons, followed by breast reconstruction. Axillary lymph node sentinel (SLNB) was performed before breast surgery and reconstruction. Antibiotic prophylaxis is performed in implant-based breast reconstruction. The implant is placed at the same time as the mastectomy. In case of delayed breast reconstruction, reconstruction surgery will begin in the period after mastectomy and oncological therapy. The minimum time after which delayed breast reconstruction can be performed is three months after the end of adjuvant chemotherapy or six months from the end of radiation therapy after mastectomy. The timing of breast reconstruction will depend on the type and duration of oncologic therapy. The treatment of patients is discussed by a multidisciplinary team consisting of plastic surgeons, radiologists, oncologists, and psychologists who are dedicated to the care of breast cancer patients.

All patients were treated according to the procedure for diagnosis and treatment of patients with breast cancer at the University Hospital Osijek.

Criteria for inclusion in this study were patients 18 years of age or older, patients with confirmed pathohistological diagnosis of stage I and stage II breast cancer and who underwent immediate or delayed unilateral breast reconstruction after mastectomy, or underwent mastectomy without reconstruction, patients without distant metastases, after completed oncological treatment, patients with good general psychophysical condition, and patients who speak and read the Croatian language.

Exclusion criteria included the development of malignant disease in the contralateral breast, the presence of distant metastases, or the occurrence of other major life changes at the time of the study that could affect psychosocial well-being. Patients who had serious psychiatric and psychotic illnesses, patients without follow-up records, and those unable to communicate in Croatian were also excluded from the study.

### 2.1. Sample Size Calculation

To observe significant differences in numerical variables between the three independent groups of subjects, with a significance level of 0.05 and a strength of 0.8, the minimum required sample size was 78 subjects.

### 2.2. Measuring

#### Health Questionnaire SF-36 and SF-6D

Patients who agreed to participate in this study completed the SF-36 (short form health survey) questionnaire on demographic data (age, education, marital status, employment status, physical appearance) and clinical variables were collected from medical records (surgery, breast reconstruction time, type of oncology therapy, clinical stage of cancer-PCD, SLNB, postoperative complications, comorbidities) during a visit to a plastic surgeon in a plastic surgery department.

The Medical Outcome Study Short Form-36 Version 2 (SF-36v2) (Supplementary Materials S1) is a 36-item self-administered questionnaire used in various healthcare facilities to assess changes in symptoms and treatment outcomes for different patients undergoing medical interventions, and also for assessment of the general health of women after surgery and breast cancer therapy. The generic measure of the health questionnaire consists of the following eight subscales: physical functioning (PF), physical role (RP), physical pain (BP), general health (GH), vitality (VT), social functioning (SF), emotional role (RE), and mental health (MH). Each concept can be rated from 0 to 100, with a higher score subjectively indicating better health. The reliability and validity of this instrument have been confirmed in studies for various patients undergoing treatment. The Croatian version of the SF-36 questionnaire was used and validated in Croatia [28,29,30,31].

The questionnaire provides a complete assessment of total QOL, Physical Component Summary (PCS) and Mental Component Summary (MCS). The PF, RP, BP, and GH subscales make up the physical component summary (PCS); the subscales VT, SF, RE, and MH form a summary of the mental component (MCS). The study with the questionnaire The Medical Outcome Study Short Form-36 (SF-36) was conducted in Croatia on women with breast cancer [32].

The Short Form 6 Dimension (SF-6D) is a multi-attribute utility instrument derived from the Short-Form 36 Health Survey Version 2 (SF-36v2) quality of life questionnaire. The SF-6Dv2 describes health on 6 dimensions: physical functioning (PF), role limitations (RL), social functioning (SF), pain, mental health (MH), and vitality (VT); 5–6 severity levels, therefore describe 18,750 health states. SF-6Dv2 is an improved version of SF-6D, one of the most widely used generic measures of health for the calculation of quality-adjusted life years. In the economic evaluation of health interventions, the quality adjusted life year (QALY) can be used to measure outcomes. The QALY combines length and quality of life into a single figure. The quality aspect (or utility value) is anchored on a 0 (dead) to 1 (full health) scale [33,34]. A common measure is developed to enable comparisons across different areas of healthcare. This measure ideally encapsulates the impact of a treatment on a patient’s length of life and also the impact on their health-related quality of life, which is a key indicator of treatment outcomes. The quality-adjusted life-year (QALY) has been developed in order to capture both of these impacts and is widely used in health economics as a summary measure of health outcome, which can inform healthcare resource allocation decisions [35].

Patients who underwent a mastectomy completed the questionnaire at a check-up at a plastic surgery clinic 1 month after surgery. The time we chose for the survey was the time of women’s recovery from surgery and changes in quality of life caused by the diagnosis of breast cancer. The women also continued oncological treatment and follow-up at the Oncology Clinic. Women who decide on subsequent delayed breast reconstruction can turn to plastic surgery to arrange secondary reconstruction (none in this study).

Patients who underwent immediate breast reconstruction completed the questionnaire at a check-up at a plastic surgery clinic 3–6 months after surgery. We decided on this period because of women who are still in the process of nipple reconstruction, nipple tattooing, or secondary correction of the other breast.

Patients who underwent delayed breast reconstruction completed the questionnaire at a Plastic Surgery Clinic 12–18 months after surgery. We decided on this period because 12–18 months have passed since the primary mastectomy, but 3–6 months after the secondary or delayed reconstruction.

### 2.3. Statistical Methods

Categorical data are presented in absolute and relative frequencies. The normality of the distribution of numerical variables was tested by the Shapiro-Wilk test. Numerical data are described by the median and by the interquartile range bounds. Differences in numerical variables between three or more independent groups were tested by the Kruskal-Wallis test (post hoc Conover). We evaluated the internal reliability of the scales via the Cronbach Alpha coefficient. All *p* values are two-sided. The significance level was set to Alpha = 0.05. The statistical program MedCalc^®^ Statistical Software version 19.6 [36]. was used for the statistical analysis.

## 3. Results

The study was conducted on 79 patients, with a median age of 51 years (interquartile range of 46 to 58 years) ranging from 34 to 68 years. The median age at which they became ill was 49 years (interquartile range of 44 to 56 years), ranging from 33 to 66 years, 37 patients underwent immediate breast reconstruction, and 17 patients underwent delayed reconstruction after mastectomy (Table 1).

Patients who underwent a mastectomy had the lowest scores in the domain of pain 18.8 (6.3–31.3) in physical functioning and limitation due to emotional difficulties 16.7 (8.3–33.3) in mental health. In immediate breast reconstruction, patients rated better physical health (*p* < 0.001), while patients who underwent delayed breast reconstruction rated their mental health worse (*p* < 0.001) (Table 2).

The results showed that mastectomy had worse results in economic viability than immediate and delayed breast reconstruction. Immediate and delayed breast reconstruction did not have significant differences in economic viability (Table 3).

## 4. Discussion

The results of our study showed significant differences in the quality of life in our patients who underwent a mastectomy without breast reconstruction compared to patients who underwent breast reconstruction after mastectomy. Lower quality of life was assessed by patients who underwent mastectomy only in all subscales of physical and mental functioning after breast cancer surgery compared to patients who underwent immediate or delayed breast reconstruction measured by the SF-36 questionnaire, which is consistent with previously published studies [37]. In contrast, a study in Brazil showed that quality of life was not significantly better in the reconstruction group than in the non-reconstruction group, and there was no significant difference in the quality of life between women with immediate and delayed reconstruction. That study showed that the satisfaction of patients with the operated breast, reconstructed or not, is more important in the quality of life than whether the breast was reconstructed or not [38]. Dauplat et al. (2017), found that mastectomy followed by reconstruction preserved the QOL, but only if reconstruction was for certain types of patients, such as young age [39]. Patients who underwent mastectomy evaluated lower results in the domains of physical health subscales in physical functioning, which refers to problems in everyday life that are related to limitations due to physical difficulties that occur in patients after surgery and cause problems such as bathing, dressing, housekeeping, work, stair climbing, and leisure activities [40].

The incidence of pain after mastectomy mainly includes items on the extent to which pain affected daily life activities, ability to walk, sleep, and mood, and was associated with lower HRQOL among breast cancer patients. These problems are pronounced in the early period after mastectomy when women often feel tense and emotionally irritable, which affects their mental health [41]. In the study in Poland, most study patients, underwent breast cancer surgery between 1 and 10 years before the study (68%), and in 20% of patients, the surgical procedure had been performed more than 10 years before they entered the study. Most study patients did not undergo breast reconstruction after mastectomy (76%). The majority of study patients responded that they experienced frequent (32%) or occasional (20%) pain in their ipsilateral upper limb [42]. This shows that pain can persist for many years after a mastectomy and affect the quality of life. When it comes to the perception of general health, patients assessed that their general health after mastectomy as poor and that they are more prone to more frequent illnesses than other people. Due to the negative impact of illness and treatment on the quality of life, women feel pain, fatigue, their body image perception changes, and there are limitations in bodily functions and a decrease in self-esteem [43].

The results of our research in the field of mental health were low in the domains of energy/vitality, limitations due to emotional difficulties, and mental health. This can be explained by the fact that the vitality subscale assesses fatigue in patients affected with chronic diseases whose quality of life is impaired, especially in breast cancer patients undergoing surgery and oncology therapy [44]. Emotional difficulties in women after a mastectomy cause them to experience different feelings of guilt, anger, and negative emotions. Some emotional changes, such as frustration and multiple conflicts, can occur during or after a period of greater tension and become mostly physical limitations [45].

Mental health plays a key role in the dynamics and self-efficacy of every person and is one of the elements for assessing the health status of society. Studies conducted in women who underwent mastectomy showed changes in body image, dissatisfaction with breast scarring, psychosocial difficulties, and deterioration of overall mental health and quality of life as a result of surgery and in accordance with the results of the study [44,46,47].

In assessing the social functioning of all three groups, patients assessed that physical health or emotional problems did not affect normal social activities in the family, with friends, neighbors, or in society that were measured at different times in our patients.

Social support is key to the survival and quality of life of patients with chronic diseases, including breast cancer patients. Social support includes informational, emotional, and instrumental support, such as going to the doctor and transportation to a support group meeting, home help, and assistance with daily activities that are key to improving positive treatment outcomes, and family members and health professionals are key sources of support. A support system for the coordination of different types of support for families and health professionals using a team-based approach can bring better social support to breast cancer patients and yield positive treatment results. [48,49].

The difference in health assessment between all three groups was not statistically significant compared to last year.

Breast reconstruction has now become an integral part of breast surgery after mastectomy because it is considered oncologically safe and aesthetically satisfactory. It should be noted that immediate breast reconstruction is increasingly recommended for all women who have undergone mastectomy for satisfaction with the appearance of the breast, as well as psychosocial, sexual, and physical well-being, which has a positive effect on quality of life [50].

In this study focusing on immediate and delayed breast reconstruction, the results showed that there was no statistically significant difference between the two groups of patients in the assessment of quality of life in the domains of the SF-36 questionnaire.

The only difference that was observed was in the domain of energy and vitality. Patients who underwent an immediate breast reconstruction evaluated higher results on a scale compared to patients who underwent delayed reconstruction. Patients who underwent an immediate reconstruction experienced less suffering and pain and had better psychosocial well-being than patients who underwent a delayed breast reconstruction. Most women were satisfied with the immediate breast reconstruction and had a better emotional well-being, aesthetic satisfaction with the body image and completion of surgical treatment [51].

In this study, most patients were younger (median age 51 years), were married, and had the support of family and partners in social and societal functioning. We can imply that the type of operation did not affect the relationship with the partners. The patients were employed and educated, and most of them assessed that their physical appearance was important and decided to have breast reconstruction. The patients reported a small number of complications in implant-based research. Complications after surgery include bleeding, hematoma, serum, and infections, and the complications after breast reconstruction depend on the type of reconstruction and the type of oncological treatment. Complications of the capsular contracture and implant extrusion are common in implant reconstruction, according to other studies [52,53,54,55,56].

Patients who underwent an immediate breast reconstruction had a better assessment of their mental health, which can be compared to a study conducted by Zang et al. (2016). The results showed that patients who underwent a mastectomy with primary breast reconstruction had better results than patients who underwent delayed breast reconstruction during psychosocial stress, poor self-image, and reduced sexual well-being and quality of life associated with health [57], while the overall physical health was better assessed by patients who underwent delayed breast reconstruction, which is in line with the results of other studies [58]. In a study conducted between immediate and delayed breast reconstruction, the results showed that delayed breast reconstruction after a mastectomy was associated with lower complication rates and provided equal satisfaction and quality of life, and delayed breast reconstruction did not appear to compromise clinical or reported outcomes of patients [59]. The oncological therapy performed in these patients also influenced the results of this study and the quality of life of these patients, which is an important factor in determining when to schedule breast reconstruction [60]. Oncological therapy was mainly related to the stage of the tumor and the type of breast reconstruction.

The SF-36 questionnaire we used in this study converted its items to QALY (using SF-6DV2 [33,34], in which age was adjusted for the quality of life, and we compared economic viability between three independent groups of patients who underwent mastectomy and immediate and delayed breast reconstruction. The results showed that mastectomy had worse results in economic viability than immediate and delayed breast reconstruction. Regarding the immediate breast reconstruction, it is the most cost-effective procedure since it is performed as a one-step surgery after mastectomy and requires one operation and one anesthesia, resulting in fewer complications and better aesthetic outcomes for the patient, as shown by other studies [15,61]. In this study, immediate and delayed breast reconstruction did not have significant differences in economic viability since patients assessed good quality of life outcomes, and complications and risk factors such as smoking, hypertension, and diabetes mellitus did not affect the results of our study.

Breast reconstruction time should be tailored to the individual needs of patients since both types of surgery result in a good quality of life [62]. Also, the interaction between the patient and the physician during the clinical consultation has a significant impact on the overall experience of the patient with breast cancer. Physicians are required to master excellent communication skills to inform patients well, improve patient selection criteria, and provide good clinical care during treatment [63].

The limitations of this study are the small sample size and short follow-up of patients in a single center. There are no results and data on the quality of life of patients before breast reconstruction, so the comparison with quality of life after surgery is impossible. There is insufficient information in the study on the reported complications of a particular operation as one of the important factors to consider when deciding on an appropriate method of breast reconstruction. There are insufficient data on preoperative chemotherapy and radiation therapy in patients who have undergone breast reconstruction.

Chemotherapy and radiation therapy before surgery are associated with the risk of postoperative complications. There is a lack of data on patient satisfaction and the results of the aesthetic appearance of reconstructive breasts, which are results of special interest to plastic surgeons and patients.

The number of patients is small, and the follow-up period of our patients was short due to the onset of the COVID-19 pandemic. In 2020, we were no longer able to follow our women because many hospitals, including ours, set restrictions on breast reconstruction in an effort to conserve resources and redirect them to patients with COVID-19. National health care system measures for controlling the spread of COVID-19 had a detrimental effect on the number of newly diagnosed breast cancer cases in Croatia. Although the formal lockdown of hospitals affected the number of newly diagnosed breast cancers, the oncology health care system has shown resilience and compensated for these effects by the end of 2020 [64].

## 5. Conclusions

The results of this study show that patients who underwent a mastectomy have a poorer assessment of their quality of life than patients who underwent an immediate or delayed breast reconstruction. In the results of this study, there is no difference in the quality of life between the patients who underwent immediate and delayed breast reconstruction.

Breast reconstruction is an important part of treatment after a mastectomy and represents good psychological, social, and emotional aspects of quality of life. The choice and type of breast reconstruction is an important selection criterion in agreement with the patient and the plastic surgeon. Future research requires a larger population of patients with a long-term follow-up to determine the quality of life outcomes and select appropriate surgical procedures.

## Figures and Tables

**Table 1:** Basic characteristics of the patients.

	Number of (%) Patients
Level of education	
Elementary school	4 (5)
High school	45 (57)
College	30 (38)
Marital status	
Married	44 (55.7)
Extramarital union	13 (16.5)
Divorced	18 (22.8)
Widow	4 (5.1)
Work status	
Employed	53 (67)
Unemployed	25 (32)
Physical appearance matters	79 (100)
Breast reconstruction	
Only a mastectomy-non-reconstruction	25 (31.6)
Primary-immediate	37 (46.8)
Secondary-delayed	17 (21.5)
Have they undergone oncological therapy	
Yes	65 (82.3)
No	13 (16.5)
Unanswered	1 (1.3)
What type of oncological therapy have they received	
chemotherapy	25 (37.8)
radiotherapy (radiation)	32 (48.4)
hormon therapy	9 (13.8)
Clinical phase-PHD	
I	40 (50.6)
II	39(49.4)
Sentinel lymph nodes	72(91)
Complications	5 (6.3)
Smoking	36 (45.6)
Hipertension	21 (26.6)
Diabetes	8 (10.1)

**Table 2:** Quality of life scale values (SF-36) in patients who underwent mastectomy, primary, and secondary reconstruction (Cronbach Alpha = 0.710).

Domains SF-36	Median (IQR)			*p* *
	Only a Mastectomy (n = 25) (1)	Primary-Immediate Reconstruction (n = 37) (2)	Secondary-Deleyed Reconstruction (n = 17) (3)	
Physical health				
Physical functioning	30 (22.5–37.5)	75 (60–85)	70 (30–90)	<0.001 ^†^
Restriction due to physical difficulties	18.8 (6.3–31.3)	62.5 (50–98.4)	75 (40.6–100)	<0.001 ^†^
Pain	30 (30–40)	75 (60–80)	70 (35–95)	<0.001 ^†^
Perception of general health	40 (36–52)	67 (57–82)	69.5 (44.5–82)	<0.001 ^†^
Mental health				
Energy/vitality	20 (15–25)	60 (50–75)	47.5 (31.3–78.8)	<0.001 ^†^
Social functioning	50 (50–50)	50 (50–62.5)	50 (50–50)	>0.99
Limitation due to emotional difficulties	16.7 (8.3–33.3)	75 (52.08–100)	75 (52.1–100)	<0.001 ^†^
Mental health	24 (20–32)	56 (48–72)	56 (52–73)	<0.001 ^†^
Overall physical health	31.6 (25.4–36.9)	68.8 (57.4–80.9)	74.3 (40.4–91.8)	<0.001 ^†^
Overall mental health	28.5 (24.3–32.5)	64.4 (52.0–72.1)	57.3 (43.9–72.3)	<0.001 ^†^
Changes in health compared to last year	50 (25–50)	50 (50–75)	50 (25–50)	<0.001 ^‡^

* Kruskal-Wallis test (post hoc Conover); ^†^ at the level of *p* < 0.05 significant differences in (1) vs. (2); (1) vs. (3); ^‡^ at the level of *p* < 0.05 significant differences in (1) vs. (2); (2) vs. (3).

**Table 3:** Economic value.

	Medijan (IQR)			*p* *
	Only a Mastectomy (n = 25)	Primary-Immediate Reconstruction (n = 37)	Secondary-Delayed Reconstruction (n = 17)	
QALY SF	0.354 (0.313–0.436)	0.427 (0.359–0.511)	0.427 (0.348–0.539)	0.08

* Kruskal-Wallis test.

## Data Availability

Data available on request from the authors.

## Supplementary Materials

The following supporting information can be downloaded at: https://www.mdpi.com/article/10.3390/ijerph19148546/s1, S1: SF-36^®^ Health Survey Update.
