# Supplementary material for: Impact of Immediate and Delayed Breast Reconstruction on Quality of Life of Breast Cancer Patients
Source: Int J Environ Res Public Health. 2022 Jul 13;19(14):8546. doi: 10.3390/ijerph19148546 (PMC9323042; doi:10.3390/ijerph19148546)
Supplement: Supplementary file 1 [file ijerph-19-08546-s001.zip › ijerph-1777061-supplementary.pdf]

SF-36® Health Survey Update

John E. Ware, Jr., Ph.D.

SF-36 Literature
Construction of the SF-36
Version 2.0
Psychometric Considerations
Translations
Discussion

The SF-36 is a multi-purpose, short-form health survey with only 36 questions. It yields an 8-scale profile of functional health and well-being scores as well as psychometrically-based physical and mental health summary measures and a preference-based health utility index. It is a generic measure, as opposed to one that targets a specific age, disease, or treatment group. Accordingly, the SF-36 has proven useful in surveys of general and specific populations, comparing the relative burden of diseases, and in differentiating the health benefits produced by a wide range of different treatments. This book chapter summarizes the steps in the construction of the SF-36; how it led to the development of an even shorter (1-page, 2-minute) survey form -- the SF-12; the improvements reflected in Version 2.0 of the SF-36; psychometric studies of assumptions underlying scale construction and scoring; how they have been translated in more than 50 countries as part of the International Quality of Life Assessment (IQOLA) Project; and studies of reliability and validity.

SF-36 Literature

The experience to date with the SF-36 has been documented in nearly 4,000 publications; citations for those published in 1988 through 2000 are documented in a bibliography covering the SF-36 and other instruments in the "SF" family of tools (Turner-Bowker, Bartley, & Ware, 2002). The most complete information about the history and development of the SF-36, its psychometric evaluation, studies of reliability and validity, and normative data is available in the first of three SF-36 user's manuals (Ware, Snow, Kosinski, & Gandek, 1993). This information was also summarized in the first two peer-reviewed articles about the SF-36 (Ware & Sherbourne, 1992; McHorney, Ware, & Raczek, 1993). A second manual documents the development and validation of the SF-36 physical and mental component summary measures and presents norms for those measures (Ware, Kosinski, & Keller, 1994; Ware, Kosinski, & Dewey, 2000). These user's manuals have been updated to include more up-to-date norms and other findings and to document the much improved Version 2.0 (SF-36v2), which are discussed below (Ware et al., 2000; Ware & Kosinski, 2001) A fourth manual, first published in 1995 (Ware, Kosinski, & Keller, 1995) and recently updated (Ware, Kosinski, Turner-Bowker, & Gandek, 2002) presents similar information for the SF-12 Health Survey, an even shorter version constructed from a subset of 12 SF-36 items.

One of the most complete independent accounts of the development of the SF-36 along with a critical commentary is offered by McDowell and Newell (1996). More recently, the SF-36 was judged to be the most widely evaluated generic patient assessed health outcome measure in a bibliographic study of the growth of "quality of life" measures published in the *British Medical Journal* (Garratt, Schmidt, Mackintosh, & Fitzpatrick, 2002). Additional information about the SF-36 literature and a community forum for discussing old and new publications and the interpretation of results are available on the SF-36 web page (http://www.sf-36.com).

The usefulness of the SF-36 in estimating disease burden and comparing disease-specific benchmarks with general population norms is illustrated in articles describing

more than 200 diseases and conditions. Among the most frequently studied diseases and conditions, with 50 or more SF-36 publications each, are: arthritis, back pain, cancer, cardiovascular disease, chronic obstructive pulmonary disease, depression, diabetes, gastro-intestinal disease, migraine headache, HIV/aids, hypertension, irritable bowel syndrome, kidney disease, low back pain, multiple sclerosis, musculoskeletal conditions, neuromuscular conditions, osteoarthritis, psychiatric diagnoses, rheumatoid arthritis, sleep disorders, spinal injuries, stroke, substance abuse, surgical procedures, transplantation, and trauma (Turner-Bowker et al., 2002).

Translations of the SF-36 have been the subject of more than 500 publications involving investigators in 22 countries. Ten or more studies have been published from 13 countries.

Construction of the SF-36

The SF-36 was constructed to satisfy minimum psychometric standards necessary for group comparisons. The eight health concepts were selected from 40 included in the Medical Outcomes Study (MOS) (Stewart & Ware, 1992). Those chosen represent the most frequently measured concepts in widely-used health surveys and those most affected by disease and treatment (Ware et al., 1993; Ware, 1995). The questionnaire items selected also represent multiple operational indicators of health, including: behavioral function and dysfunction, distress and well-being, objective reports and subjective ratings, and both favorable and unfavorable self-evaluations of general health status (Ware et al., 1993).

Most SF-36 items have their roots in instruments that have been in use since the 1970’s and 1980’s (Stewart & Ware, 1992), including items from: the General Psychological Well-Being Inventory (GPWBI) (Dupuy, 1984); various physical and role functioning measures (Patrick, Bush, & Chen, 1973; Hulka & Cassel, 1973; Reynolds, Rushing, & Miles, 1974; Stewart, Ware, & Brook, 1981); the Health Perceptions Questionnaire (HPQ) (Ware, 1976); and other measures that proved to be useful during the Health Insurance Experiment (HIE) (Brook, Ware, Davies-Avery, Stewart, Donald, Rogers, Williams, & Johnston, 1979). MOS researchers selected and adapted questionnaire items from these and other sources, and developed new measures for a 149-item Functioning and Well-Being Profile (FWBP) (Stewart & Ware, 1992). The FWBP was the source for questionnaire items and instructions adapted for use in the SF-36. The SF-36 was first made available in a “developmental” form in 1988 and in “standard” form in 1990 (Ware, 1988; Ware & Sherbourne, 1992). As documented elsewhere (Ware et al., 1993), the standard form eliminated more than one-fourth of the words contained in MOS versions of the 36 items and also incorporated improvements in item wording, format and scoring.

SF-36v2™ Health Survey
(Version 2.0)

In 1996, Version 2.0 of the SF-36 (SF-36v2) was introduced, to correct deficiencies identified in the original version. Those improvements, which are documented in the SF-36v2 user’s manual (Ware et al., 2000), were implemented after careful study using both qualitative and quantitative methods. Briefly, the SF-36v2 improvements include:

- improvements in instructions and questionnaire items to shorten and simplify the wording and make it more familiar and less ambiguous;
- an improved layout for questions and answers in the self-administered forms that makes it easier to read and complete, and that reduces missing responses;
- greater comparability with translations and cultural adaptations widely-used in the U.S. and in other countries;

- five -level response choices in place of dichotomous response choices for seven items in the two role functioning scales; and,
- five-level (in place of six-level) response categories to simplify items in the Mental Health (MH) and Vitality (VT) scales.

These and other improvements are briefly explained below.

Layout

All responses to questions in Version 2.0 are printed in a left-to-right (also referred to as "horizontal") format, rather than with the mixture of horizontal and vertical listings of response choices that were printed below questions in the MOS and in the original SF-36. Mixed formats of response choices confuse respondents and cause missing and inconsistent responses, particularly among the elderly. Other improvements include more consistent use of indenting, numbering of instructions, deletion of useless item labels, and a simpler formatting of boxes that are checked by respondents.

Type-size and Bolding

A larger type size has been adopted throughout. Only instructions, as opposed to response choices, are bolded to simplify the "look and feel" of Version 2.0. These and other refinements were adopted on the basis of lessons learned in health care and from surveys in other fields.

Wording Changes

Evidence from numerous focus group studies, formal cognitive tests, and from empirical studies in more than a dozen countries support the improvements in item wording and the changes in some terms used to identify health concepts adopted in Version 2.0. These improvements make the English-language SF-36 easier to understand and administer as well as making it more objective. Version 2.0 is also more comparable with translations of the SF-36. Because most of the improvements in item wording were developed during the process of translating and adapting the SF-36 for use in other countries during the International Quality of Life Assessment (IQOLA) Project, Version 2.0 is sometimes referred to as the "international version".

Five-Choice Response Scales

There is considerable empirical evidence that the Version 2.0 five-level response scales substantially improve the two SF-36 role functioning scales. Version 2.0 response scales extend the range measured and greatly increase score precision without increasing respondent burden. Specifically, Version 2.0 achieves a four-fold increase in the number of levels defined by both role scales, a substantially smaller standard deviation, and substantially reduces the percentage of respondents who score at both the ceiling and floor for both role scales. The elimination of one of the six response choices ("a good bit of the time") from the MH and VT items was based on the finding that this response choice is not consistently ordered between adjacent categories in studies of item responses in Version 1.0 or in translations of the SF-36. Eliminating this choice simplified the format of the form with little or no loss of information.

Scoring and Norms

With the release of SF-36v2, norms were updated using data from the 1998 National Survey of Functional Health Status (NSFHS) and norm-based scoring (NBS) algorithms were introduced for all eight scales (Ware et al., 2000). NBS, which employs a linear T-score transformation with mean = 50 and standard deviation = 10, makes it possible

to meaningfully compare scores for the eight-scale profile and the physical and mental summary measures in the same graph. SF-36v2 scoring software also yields less biased estimates of missing responses and makes it possible to estimate scores for more respondents with incomplete data (Kosinski, Bayliss, Bjorner, & Ware, 2000).

Comparability of Results

To make Version 1.0 easier to interpret and directly comparable to published results based on Version 2.0, cross-sectional and longitudinal norms for general and specific populations were re-estimated for Version 1.0 using NBS for all eight scales and for the two summary measures. Further, national calibration studies were fielded in the U.S. in 1998 and 1999 to evaluate the effect of all improvements and to assure the comparability of average scores across Versions 1.0 and 2.0 (Ware et al., 2000).

Acute (1-week recall) Form

The SF-36 is now available in both standard (4-week) and acute (1-week) recall versions. The more recently developed acute form was designed for applications in which health status would be measured weekly or biweekly. It was created by changing the recall period for six of the eight scales [Role-Physical (RP), Bodily Pain (BP), VT, Social Functioning (SF), Role-Emotional (RE) and MH] from "the past four weeks" to "the past week". Two scales, Physical Functioning (PF) and General Health (GH) do not have a recall period; the items and instructions for these scales are identical across acute and standard forms.

The rationale behind a form with a 1-week recall period was that it would be more sensitive to recent changes in health status. This hypothesis was tested by comparing results for both the 1-week and original 4-week recall forms administered three times during a clinical trial of treatments for asthma (Keller, Bayliss, Ware, Hsu, Damiano, & Goss, 1997). As hypothesized, answers to SF-36 questions with a 1-week recall period tended to be more responsive to recent changes in disease state as estimated using several clinical criteria defining the severity of asthma. For example, changes in acute (1-week recall) SF-36 scale scores were generally more highly related to 1-week changes in asthma severity. Of some concern, from a normative perspective, the study also revealed higher mean scores for the acute version scales in comparison with the standard form scales. One explanation offered was a lower prevalence of negative events during the shorter recall period defined by the acute form. If so, this potential difference in mean scores would have implications for the norm-based interpretation of acute form scores. However, the findings from this one clinical trial were not replicated during the 1998 norming of the acute and standard forms in the general U.S. population (Ware et al., 2000).

Psychometric Considerations

SF-36 Measurement Model

Figure 1 illustrates the taxonomy of items and concepts underlying the construction of the SF-36 scales and summary measures. The taxonomy has three levels: (1) items; (2) eight scales that aggregate 2-10 items each; and, (3) two summary measures that aggregate scales. All but one of the 36 items (self-reported health transition) are used to score the eight SF-36 scales. Each item is used in scoring only one scale.

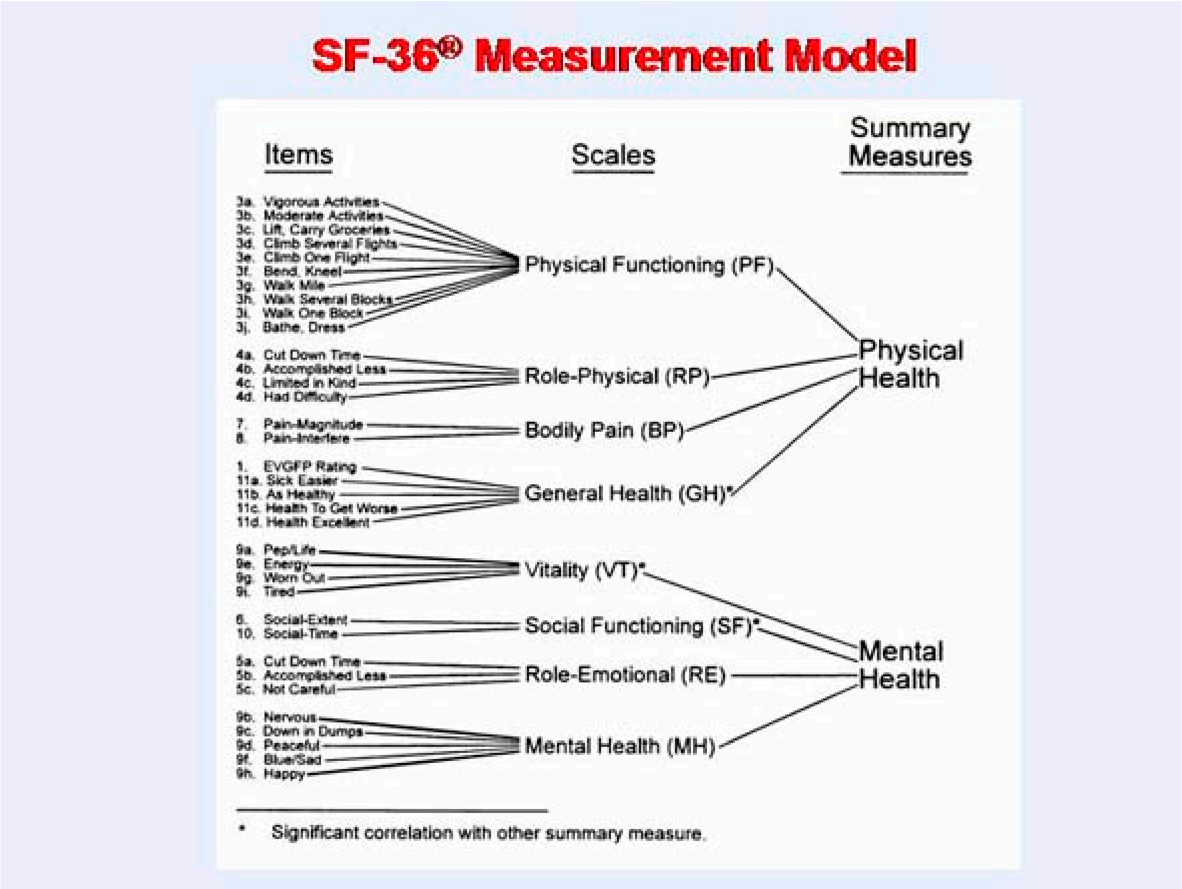

The eight scales are hypothesized to form two distinct higher-ordered clusters due to the physical and mental health variance that they have in common. Factor analytic studies have confirmed physical and mental health factors that account for 80-85% of the reliable variance in the eight scales in the U.S. general population (Ware et al., 1994), among MOS patients (McHorney et al., 1993; Ware et al., 1994), and in general populations in Sweden (Sullivan, Karlsson, & Ware, 1995) and the UK (Ware et al., 1994). As of 1998, these studies had been replicated in more than a dozen countries (Ware, Kosinski, Gandek, Aaronson, Alonso, Apolone, Bech, Brazier, Bullinger, Kaasa, Leplege, Prieto, & Sullivan, 1998; Fukuhara, Ware, Kosinski, Wada, & Gandek, 1998).

Three scales (PF, RP, BP) correlate most highly with the physical component and contribute most to the scoring of the Physical Component Summary (PCS) measure (Ware et al., 1994). The mental component correlates most highly with the MH, RE, and SF scales, which also contribute most to the scoring of the Mental Component Summary (MCS) measure. Three of the scales (VT, GH, and SF) have noteworthy correlations with both components.

The importance of these findings is illustrated below in the discussion of empirical validity. Specifically, scales that load highest on the physical component are most responsive to treatments that change physical morbidity, whereas scales loading highest on the mental component respond most to drugs and therapies that target mental health.

Scaling and Scoring Assumptions

A major objective in constructing the SF-36 was achievement of high psychometric standards. Guidelines for testing were derived from those recommended for use in validating psychological and educational measures by the American Psychological Association, the American Education Research Association, and the National Council on

Measurement in Education (APA, 1974). Extensive psychometric testing has been conducted on the SF-36 in the United States (McHorney, Ware, Lu, & Sherbourne, 1994; Garratt, Ruta, Abdalla, Buckingham, & Russell, 1993; Jenkinson, Coulter, & Wright, 1993; Wagner, Keller, Kosinski, Baker, Jacoby, Hsu, Chadwick, & Ware, 1995), other countries (Sullivan, Karlsson, & Ware, 1994; Rampal, Martin, Marquis, Ware, & Bonfils, 1994; Sullivan et al., 1995; Bullinger, 1995; McCallum, 1995). Using the same tests of scaling and scoring assumptions that were used in developing the SF-36, results have been compared across general population studies in 10 countries (Gandek & Ware, 1998).

On the strength of favorable results from tests to date, nearly all studies have used the method of summated ratings and standardized SF-36 scoring algorithms documented elsewhere (MOT, 1991; Ware et al., 1993). This method assumes that items shown in the same scale in Figure 1 can be aggregated without score standardization or item weighing. Standardization of items within a scale was avoided by selecting or constructing items with roughly equivalent means and standard deviations. Weighting was avoided by using equally representative items (that is, items with roughly equivalent relationships to the underlying scale dimension). All items have been shown to correlate substantially (greater than 0.40, corrected for overlap) with their hypothesized scales with rare exceptions (McHorney et al., 1994; Ware et al., 1993).

More recent studies using item response theory (IRT) have shown strong linear associations between the original SF-36 simple summated ratings scores and those derived from IRT models, except at the extremes, as would be expected (Haley, McHorney, & Ware, 1994; McHorney, Haley, & Ware, 1997; Raczek, Ware, Bjorner, Gandek, Haley, Aaronson, Apolone, Bech, Brazier, Bullinger, & Sullivan, 1998). Results from these IRT studies have also suggested that improvements in scales and scoring algorithms are possible, especially for the PF scale. These models have also revealed substantial increases in the range of scale levels measured by both of the SF-36v2 role functioning scales in comparison with the original versions of those scales (Ware et al., 2000). Among the practical implications are greater score precision and reduced concentrations of scores at the "ceiling" and "floor".

Reliability and Confidence Intervals

The reliability of the eight scales and two summary measures has been estimated using both internal consistency and test-retest methods. With rare exceptions, published reliability statistics have exceeded the minimum standard of 0.70 recommended for measures used in group comparisons in more than 25 studies (Tsai, Bayliss, & Ware, 1997); most have exceeded 0.80 (McHorney et al., 1994; Ware et al., 1993). Reliability estimates for physical and mental summary scores usually exceed 0.90 (Ware et al., 1994). A review of the first15 published studies revealed that the median reliability coefficients for each of the eight scales was equal or greater than 0.80 except for SF, which had a median reliability across studies of 0.76 (Ware et al., 1993). In addition, a reliability of 0.93 has been reported for the MH scale using the alternate forms method, suggesting that the internal-consistency method underestimated the reliability of that scale by about three percent (McHorney & Ware, 1995).

The trends in reliability coefficients for the SF-36 scales and summary measures summarized above have also been replicated across 24 patient groups differing in socio-demographic characteristics and diagnoses (Ware et al., 1993; Ware et al., 1994); McHorney et al., 1994). While studies of subgroups indicate slight declines in reliability for more disadvantaged respondents, reliability coefficients consistently exceeded recommended standards for group level analysis. Reliability estimates consistent with these trends have been published in more than 200 studies, results from more than 30 test-retest studies have also been summarized (Turner-Bowker et

al., 2002).

Standard errors of measurement, 95% confidence intervals for individual scores, and distributions of change scores from test-retest and one-year stability studies have been published for the eight SF-36 scales and for the two summary scores (Brazier, Harper, Jones, O'Cathain, Thomas, Usherwood, & Westlake, 1992; Ware et al., 1993; Ware et al., 1994). Confidence intervals around individual scores are much smaller for the two summary measures than for the eight scales (+/- 6-7 points versus +/- 13-32 points, respectively) (Ware et al., 1994). For purposes of the Medicare Health Outcomes Survey – a very large federal effort to monitor health outcomes across health care plans serving the Medicare population – psychometrically-based standards have been established for SF-36 scores used to classify changes (better, same or worse) in physical (PCS) and mental (MCS) component summary scores (NCQA, 2002). Estimates of sample sizes required to detect differences in average scores of various magnitudes have been documented for five different study designs for each of the eight scales and for the two summary measures (Ware et al., 1993; Ware & Kosinski, 2001; Ware et al., 1994).

Validity

Studies of validity generally support the intended meaning of high and low SF-36 scores as documented in the original user's manuals (Ware et al., 1993; Ware et al., 1994). Because of the widespread use of the SF-36 across a variety of applications, evidence from many types of validity research is relevant to these interpretations. Studies to date have yielded content, concurrent, criterion, construct, and predictive evidence of validity.

The content validity of the SF-36 has been compared to that of other widely used generic health surveys (Ware et al., 1993; Ware, 1995). Systematic comparisons indicate that the SF-36 includes eight of the most frequently measured health concepts. Among the content areas included in widely-used surveys, but not included in the SF-36, are; sleep adequacy, cognitive functioning, sexual functioning, health distress, family functioning, self-esteem, eating, recreation/hobbies, communication, and symptoms/problems that are specific to one condition. Symptoms and problems that are specific to a particular condition are not included in the SF-36 because the SF-36 is a generic measure.

To facilitate the evaluation of concepts not included, the SF-36 users" manuals include tables of correlations between the eight scales and the two summary measures and 32 measures of other general concepts (Ware et al., 1993; Ware et al., 1994), as well as 19 specific symptoms. SF-36 scales correlate substantially (r=0.40 or greater) with most of the omitted general health concepts and with the frequency and severity of many specific symptoms and problems. A noteworthy exception is sexual functioning, which correlates relatively weakly with SF-36 scales and is a good candidate for inclusion in questionnaires that supplement the SF-36.

Because most SF-36 scales were constructed to reproduce longer scales, attention was initially given to how well the short-form versions perform in empirical tests relative to the full-length versions. Relative to the longer MOS measures they were constructed to reproduce, SF-36 scales have been shown to achieve about 80-90% of their empirical validity in studies involving physical and mental health "criteria" (McHorney et al., 1993).

The validity, and therefore the interpretation, of each of the eight scales and the two summary measures has been shown to differ markedly, as would be expected from factor analytic studies of their construct validity (see Figure 2) (McHorney et al., 1993; Ware et al., 1994; Ware, Kosinski, Bayliss, McHorney, Rogers, & Raczek, 1995).

Specifically, the MH, RE, and SF scales and the MCS summary measure have been shown to be the most valid of the SF-36 scales as mental health measures. This pattern of results has been replicated in both cross-cultural and longitudinal tests using the method of known-groups validity. The PF, RP, and BP scales and the PCS summary have been shown to be the most valid SF-36 scales for measuring physical health. Criteria used in the known-groups validation of the SF-36, which include accepted clinical indicators of diagnosis and severity of depression, heart disease, and other conditions, are well documented in peer-reviewed publications and in the two users" manuals (Kravitz, Greenfield, Rogers, Manning, Zubkoff, Nelson, Tarlov, & Ware, 1992; McHorney et al., 1993; Ware et al., 1993; Ware et al., 1994; Ware et al., 1995).

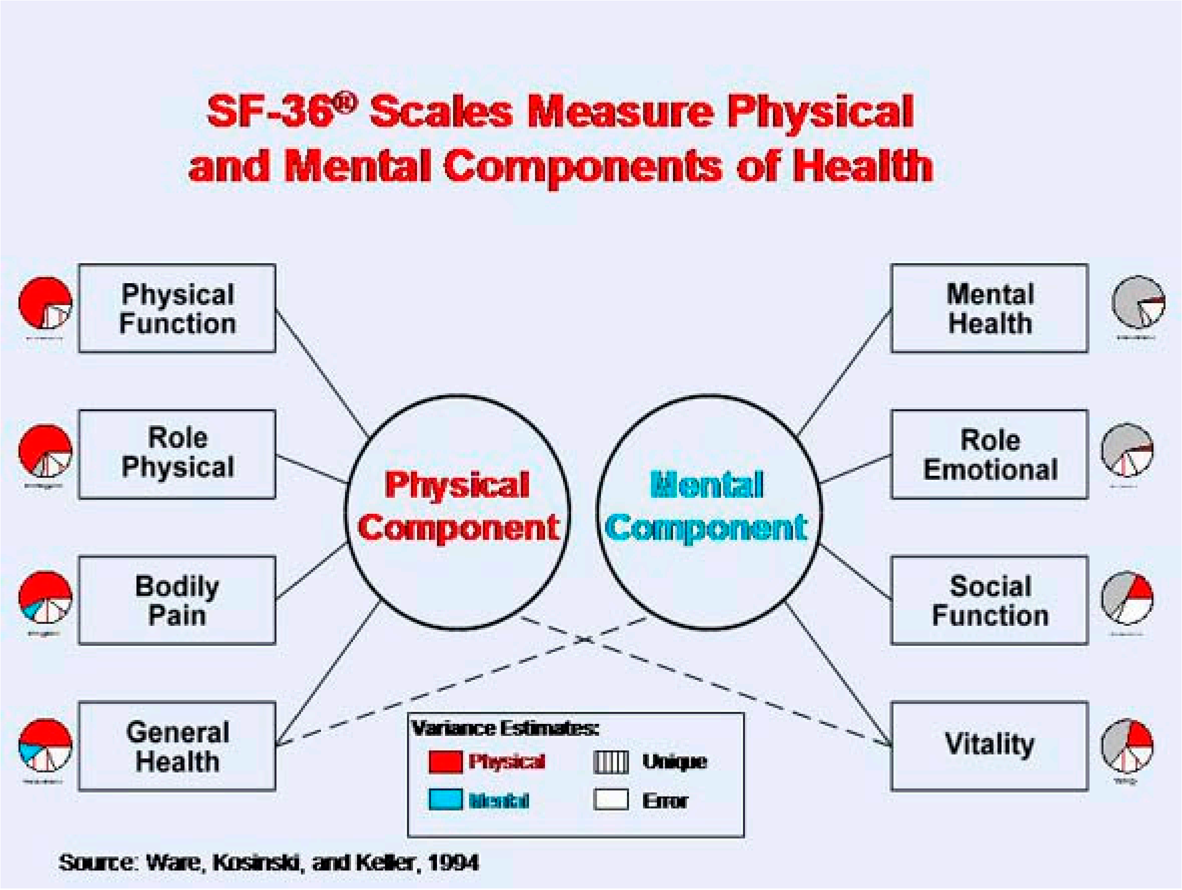

The MH scale has been shown to be useful in screening for psychiatric disorders (Berwick, 1991; Ware et al., 1994), as has the MCS summary measure (Ware et al., 1994). For example, using a cutoff score of 42, the MCS had a sensitivity of 74% and a specificity of 81% in detecting patients diagnosed with depressive disorder (Ware et al., 1994).

Relative to other published measures, SF-36 scales have performed well in most tests published to date (Weinberger, Samsa, Hanlon, Schmader, Doyle, Cowper, Uttech, Cohen, Feussner, 1991; Brazier et al., 1992; Kantz, Harris, Levitsky, Ware, & Davies, 1992; Krousel-Wood & Re, 1994; Krousel-Wood, McCune, Abdoh, & Re, 1994). As cited in the SF-36 bibliography (Turner-Bowker et al., 2002), studies have compared the SF-36 with 225 other measures. Predictive studies of validity have linked SF-36 scales and summary measures to utilization of health care services (Ware et al., 1994), the clinical course of depression (Wells, Burnam, Rogers, Hays, & Camp, 1992; Beusterien, Steinwald, & Ware, 1996), loss of job within one year (Ware et al., 1994), 180-day survival (Rumsfield, MaWhinney, McCarthy, Shroyer, Villa Nueva, O'Brien, Moritz, Henderson, Grover, Sethi, & Hammerstein, 1999) and five-year survival (Ware et al., 1994).

Results from clinical studies comparing scores for patients before and after treatment have largely supported hypotheses about the validity of SF-36 scales based on psychometric studies. For example, clinical studies have shown that three of the scales (PF, RP, and BP) with the most physical factor content (Figure 2) tend to be most responsive to the benefits of knee replacement (Kantz et al., 1992), hip replacement (Kantz et al., 1992; Lansky, Butler, & Waller, 1992), and heart valve surgery (Phillips & Lanky, 1992). In contrast, the three scales with the most mental factor content (MH, RE, and SF) in factor analytic studies have been shown to be most responsive in comparisons of patients before and after recovery from depression (Ware et al., 1995); change in the severity of depression (Beusterien et al., 1996); as well as drug treatment and interpersonal therapy for depression (Coulehan, Schulberg, Block, Madonia, & Rodrigues, 1997).

The discovery that 80-85% of the reliable variance in the eight SF-36 scales led to the construction of psychometrically-based physical and mental health summary measures. It was hoped that they would make it possible to reduce the number of statistical comparisons involved in analyzing the SF-36 (from eight to two) without substantial loss of information. In both cross-sectional and longitudinal studies reported to date, this appears to be the case (Ware et al., 1994; Ware et al., 1995). The advantages and disadvantages of analyzing the eight-scale SF-36 profile versus the two summary measures are illustrated and discussed elsewhere (Ware et al., 1994; Ware et al., 1995).

Finally, the SF-36 self-evaluated health transition item (five response categories ranging from "much better" to "much worse"), which is not used in scoring the scales or summary measures, has been shown to be useful in estimating average changes in health status during the year prior to its administration. In the MOS, measured changes in health status during a one-year follow-up period corresponded substantially, on average, to self-evaluated transitions at the end of the year. Using the 0-100 General Health Rating Index (GHRI) scale (Davies & Ware, 1981) as a "criterion", those who evaluated their health as "much better" improved an average of 13.2 points. The average change was 5.8 points for those who reported that they were "somewhat better". An average decline of -10.8 was observed for those who reported that their health was "somewhat worse" and 34.4 for those reporting "much worse". (It should be noted that the latter category had only 29 patients.) Change scores for those choosing the "about the same" category averaged 1.6 points. These results are encouraging with regard to the use and interpretation of self-evaluated transitions at the group level. Pending results from ongoing studies of the reliability of responses to the SF-36 self-evaluated transition item, it should be interpreted with caution at the individual level. Additional results and their implications are discussed elsewhere (Ware et al., 1993; Ware et al., 1994).

Administration Methods and Scoring

The SF-36 is suitable for self-administration, computerized administration, or administration by a trained interviewer in person or by telephone, to persons age 14 and older. The SF-36 has been administered successfully in general population surveys in the U.S. and other countries (Ware, Keller, Gandek, Brazier, & Sullivan, 1995), as well as to young and old adult patients with specific diseases (Ware et al., 1993; McHorney et al., 1994). It can be administered in 5-10 minutes with a high degree of acceptability and data quality (Ware et al., 1993). Indicators of data quality that have yielded satisfactory results in studies to date include very high item completion rates and favorable results for a response consistency index based on 15 pairs of SF-36 items, which is scored at the individual level (Ware et al., 1993). Computer administered and telephone voice recognition interactive systems of administration are currently being evaluated. Online administrations and scoring of SF-36 forms are

demonstrated on the Internet.

Summary Measures

Table 1 summarizes information about the eight SF-36 scales and two summary measures that is important in their use and nterpretation. The eight scales are ordered in Table 1 in terms of their factor content (i.e., construct validity) as they are in the SF-36 profile to facilitate interpretation. The first scale is PF, which has been shown to be the best all around measure of physical health; the last scale, MH is the most valid measure of mental health in studies to date (McHorney et al., 1993; Ware et al., 1993; Ware et al., 1994). Interestingly, MH and PF are the poorest measures of the physical and mental components, respectively. Scales in between are ordered according to their validity in measuring physical and mental health. The VT and GH scales have substantial or moderate validity for both components of health status and should be interpreted accordingly.

Table 1: Summary of Information about SF-36® Scales and Physical and Mental Component Summary Measures

Summary of Information about SF-36 Scales and Physical and Mental Component Summary Measures

	Correlations		Number of						Definition (% observed)	
Scales	PCS	MCS	Items	Levels	Mean	SD	Reliability	Cla	Lowest Possible Score (Floor)c	Highest Possible Score (Ceiling)c
Physical Functioning	.85	.12	10	21	84.2	23.3	.93	12.3	Very limited in performing all physical activities, including bathing or dressing (0.8%)	Performs all types of physical activities including the most vigorous without limitations due to health (38.8%)
Role-Physical (RP)	.81	.27	4	5	80.9	34.0	.89	22.6	Problems with work or other daily activities as a result of physical health (10.3%)	No problems with work or other daily activities (70.9%)
Bodily Pain	.76	.28	2	11	75.2	23.7	.90	15.0	Very severe and extremely limiting pain (0.6%)	No pain or limitations due to pain (31.9%)
General Health (GH)	.69	.37	5	21	71.9	20.3	.81	17.6	Evaluates personal health as poor and believes it is likely to get worse (0.0%)	Evaluates personal health as excellent (7.4%)
Vitality	.47	.65	4	21	60.9	20.9	.86	15.6	Feels tired and worn out all of the time (0.5%)	Feels full of pep and energy all of the time (1.5%)
Social Functioning	.42	.67	2	9	83.3	22.7	.68	25.7	Extreme and frequent interference with normal social activities due to physical and emotional problems (0.6%)	Performs normal social activities without interference due to physical or emotional problems (52.3%)
Role-Emotional (RE)	.16	.78	3	4	81.3	33.0	.82	28.0	Problems with work or other daily activities as a result of emotional problems (9.6%)	No problems with work or other daily activities (71.0%)
Mental Health (MH)	.17	.87	5	26	74.7	18.1	.84	14.0	Feelings of nervousness and depression all of the time (0.0%)	Feels peaceful, happy, and calm all of the time (0.2%)
Physical Component Summary			35	567b	50.0	10.0	.92	5.7	Limitations in self-care, physical, social, and role activities, severe bodily pain, frequent tiredness, health rated "poor" (0.0%)	No physical limitations, disabilities, or decrements in well-being, high energy level, health rated "excellent" (0.0%)

Mental Component Summary			35	493b	50.0	10.0	.88	6.3	Frequent psychological distress, social and role disability due to emotional problems, health rated "poor" (0.0%)	Frequent positive affect, absence of psychological distress and limitations in usual social/role activities due to emotional problems, health rated "excellent" (0.0%)

Note. From Ware, Kosinski, and Keller (1994).

[a]CI=95% confidence interval

[b] Numberof levels observed at baseline; scores rounded to the first decimal place (n=2,474).

[c]Percentage observed comes from general U.S. population sample.

[d] Scores for eight scales are the percentage of the total possible score achieved for each of these scales. Scores for PCS and MCS are*T-scores.*

The number of items and levels and the range of states defined by each scale are also shown in Table 1. These attributes have been linked to their empirical validity (McHorney, Ware, Rogers, Raczek, & Lu, 1992). The most precise (least coarse) scales are those with 20 or more levels (PF, GH, VT, and MH). They also define the widest range of health states and, therefore, usually produce the least skewed score distributions. The relatively coarse role disability scales (RP and RE) each measure only four or five levels across a restricted range, and therefore, usually have the most problems with ceiling and floor effects. Means and standard deviations for each of the eight scales in the general U.S. adult population are also presented. These can be used to determine whether a group or individual in question scores above or below the U.S. average. Detailed normative data including frequency distributions of scores and percentile ranks are documented in the two users" manuals (Ware et al., 1993; Ware et al., 1994). Table 1 illustrates the practical implications of a number of theoretical advantages of the PCS and MCS summary measures including reliability, as well as the number and range of levels covered.

Another very promising approach to scoring the SF-36, reported by Brazier, Roberts and Deverill in the*Journal of Health Economics* (2002), is a preference-based health utility index This index, which is labeled the SF-6D because it uses a 6-domain classification of health states (about 18,000 in all), is the first preference-based index constructed from a “psychometric” measure of health status. The SF-6D preferences can be applied to any SF-36 dataset for purposes of economic evaluation (e.g., estimation of quality-adjusted life years – QALYs).

Norm-based Scoring and Interpretation

The interpretation of results has been made much easier with the standardization of mean scores and standard deviations for all SF-36 scales. Specifically, norm-based scoring has proven to be very useful when interpreting differences across scales in the SF-36 profile and for monitoring disease groups over time. As documented elsewhere (Ware et al., 1994), linear transformations were performed to transform scores to a mean of 50 and standard deviations of 10, in the general U.S. population. This transformation achieves the same mean and standard deviation for all eight scales and for the physical and mental summary measures.

The advantages of norm-based scoring can be illustrated by comparing the SF-36 profile scored using the original 0–100 scoring algorithms based on the summated ratings method) and the norm-based scoring algorithms for a sample of asthmatic patients who participated in a clinical trial (Okamoto, Noonan, DeBoisblanc, & Kellerman, 1996). The original SF-36 0-100 scoring produced the profile shown in Figure 3. The shape of this profile – the peaks and valleys due to higher and lower scores across scales – reflect both the impact of asthma on SF-36 health concepts, as well as arbitrary differences in the ceilings and floors of the SF-36 scales. Three scales, namely GH, VT, and MH, measure relatively wide score ranges and set the ceiling relatively high by measuring very favorable levels of those health concepts (Ware et al., 1993). Other scales, such as PF and RP, assess a narrower range. The most favorable levels (scored 100 using the original SF-36 algorithms) for PF and RP represent the absence of limitations and do not extend the range into well being. Thus, the average score for each scale differs substantially across the profile for reasons that have nothing to do with asthma, using the original SF-36 0-100 scoring. The inference from the profile in Figure 3, that asthma has a greater impact on PF than on VT, is incorrect.

SF-36® Health Profile: Adults with Asthma Compared with U.S. Norm

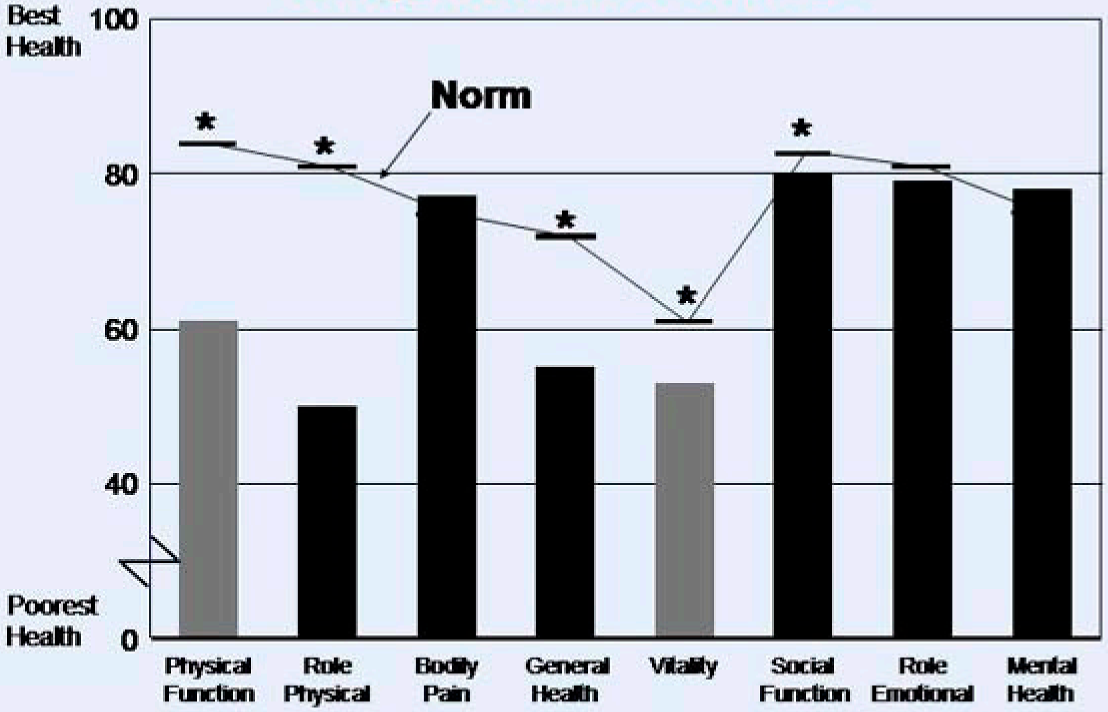

Source: Okamoto, 1996, * Norm significantly higher

Norm-based Scoring of SF-36® Profile, Adults with Asthma

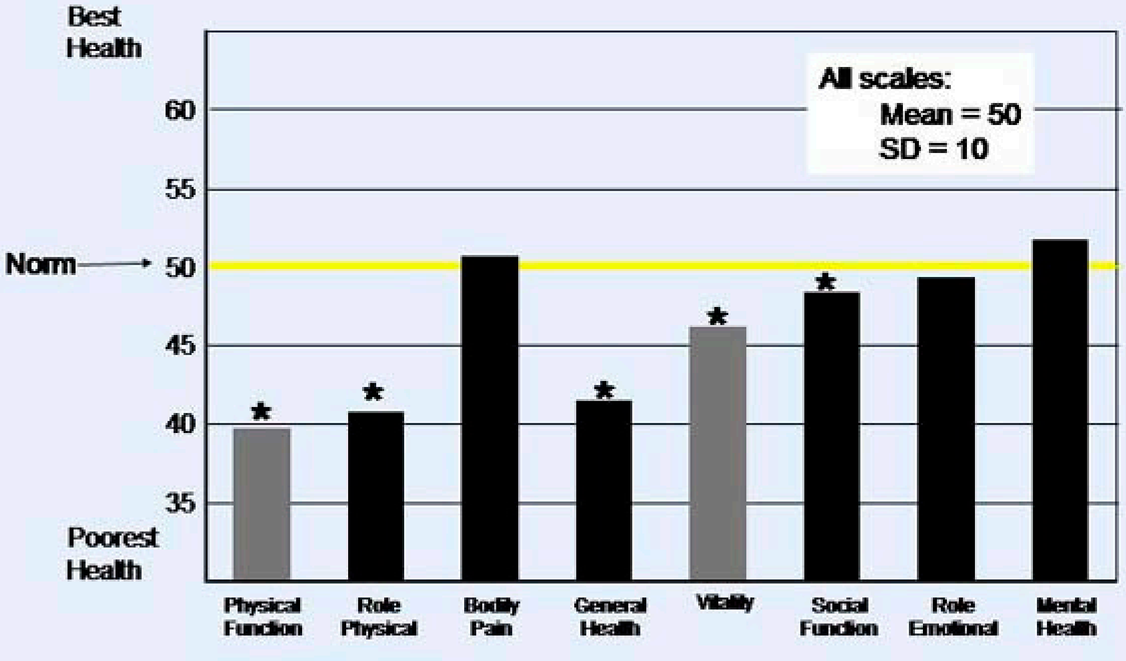

Adapted from: Okamoto, 1996

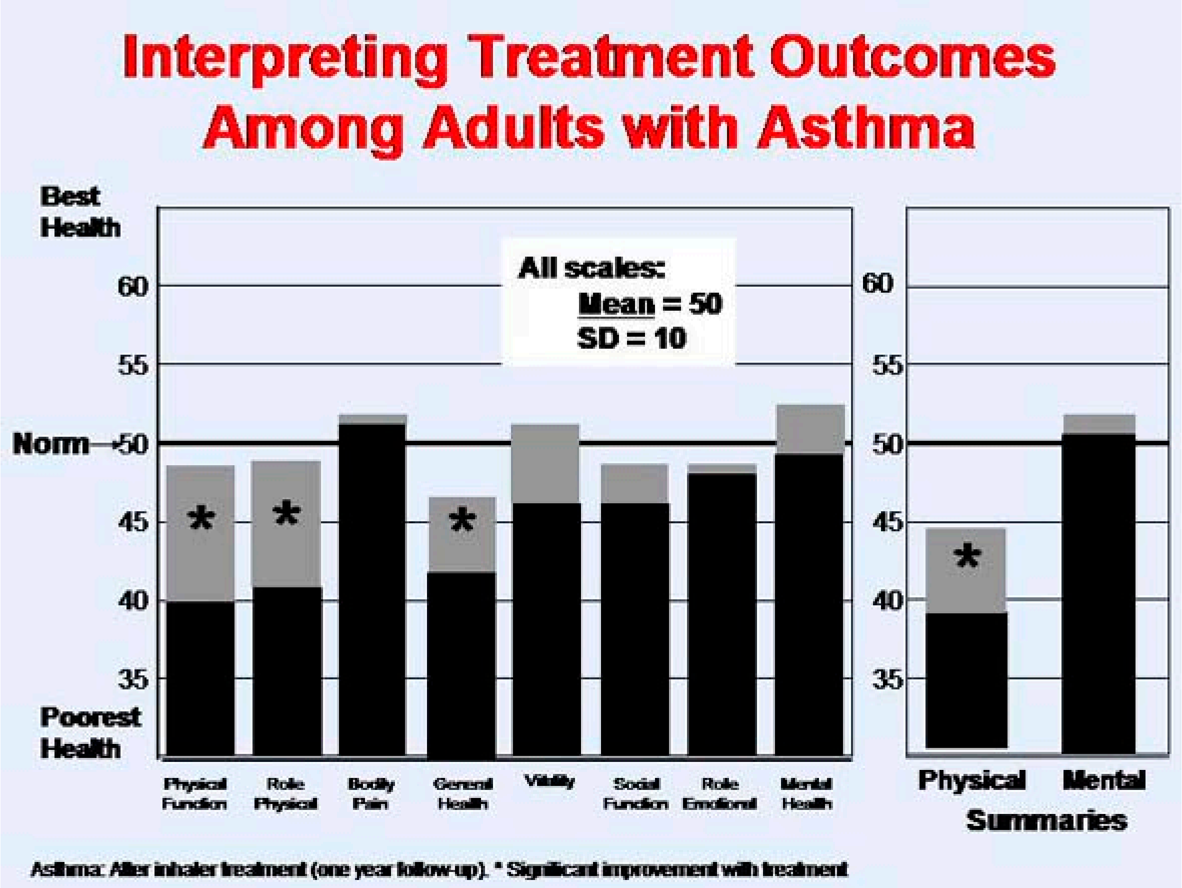

General population norms provide a much better basis for comparisons across scales (see Figure 3). For example, the PF scale averages between 80 and 90 while the VT average score is below 60 (on the 100-point score range) in the general population. In relation to these norms, the impact of asthma appears much larger on the PF scale than on the VT scale, although both are statistically significant. Using the original 0–100 scoring, these differences in norms must be kept in mind when interpreting a profile. Differences in standard deviations, which are also substantial across some scales, must also be considered for this purpose.

In norm-based scoring, each scale was scored to have same average (50) and the same standard deviation (10 points). Without referring to norms, it is clear that anytime a scale score is below 50, health status is below average, and each point is one-tenth of a standard deviation. As shown in Figure 3, with norm-based scoring, differences in scale scores much more clearly reflect the impact of the disease, in this example the impact of asthma. Clinicians can more quickly and appropriately interpret the effect of asthma on a SF-36 health profile. Because the Physical (PCS) and Mental (MCS) component summary measures take into account the correlation among the eight SF-36 scales, it is clear that asthma impacted on the physical component of health and (from the profile with five significant differences) impacted very broadly.

The application of norm-based scoring to a clinical trial of treatment effects is illustrated in Figure 3. Patients treated using an inhaler showed statistically significant improvements relative to baseline after 16 weeks of treatment on three of the eight SF-36 scales, those most closely associated with PF.

Translations

The International Quality of Life Assessment (IQOLA) Project is translating, validating, and norming the SF-36 Health Survey for use in multinational clinical trials and other international studies (Aaronson, Acquadro, Alonso, Apolone, Bucquet, Bullinger, Bungay, Fukuhara, Gandek, Keller, Razari, Sanson-Fisher, Sullivan, Wood-Dauphinee, & Ware, 1992; Ware, Gandek, & the IQOLA Project Group, 1994; Ware, Keller, Gandek, Brazier, Sullivan, & the IQOLA Project Group, 1995; Ware, Gandek, Keller, & the IQOLA Project Group, 1996; Gandek & Ware, 1998). The project, which is based at the Health Assessment Lab in Boston, began in 1991 with sponsored investigators from 14 countries: Australia, Belgium, Canada, Denmark, France, Germany, Italy, Japan, The Netherlands, Norway, Spain, Sweden, the United Kingdom (English version), and

the United States (English and Spanish versions). In addition, the SF-36 has been translated for use in more than 40 other countries, including: Argentina, Armenia, Austria, Bangladesh, Brazil, Bulgaria, Cambodia, Chile, China, Colombia, Costa Rica, Croatia, Czech Republic, Finland, Greece, Guatemala, Honduras, Hong Kong, Hungary, Iceland, Israel, Korea, Latvia, Lithuania, Mexico, New Zealand, Peru, Poland, Portugal, Romania, Russia, Singapore, Slovak Republic, South Africa, Switzerland, Taiwan, Tanzania, Turkey, the United Kingdom (Welsh), the United States (Chinese, Japanese, Vietnamese), Uruguay, Venezuela, and Yugoslavia. More than 500 publications that use translations or English-language adaptations of the SF-36 have been published.

Four major stages of activity are included. First, translation follows a standard protocol, including multiple forward and backward translations. Qualitative and quantitative methods are used to evaluate the quality of a translation and its conceptual equivalence with the original survey. Second, formal psychometric tests of scaling assumptions and scoring assumptions are conducted prior to publication of a translation. Third, data from clinical trials and other studies are being analyzed to address issues of validity and comparability across countries. Normative data are being collected in general population surveys in eleven countries for purposes of norm-based interpretation. Published norms will soon be available for 10 countries. English-language, Swedish, and Italian user's manuals are available and others are forthcoming.

Published IQOLA Project SF-36 translations and English-language adaptations are distributed royalty-free by the Health Assessment Lab. Currently, published forms include the German (Bullinger, 1995), Spanish (Alonso, Prieto, & Anto, 1995), Swedish (Sullivan et al., 1994), and Italian (Apolone, Cifani, Liberati, & Mosconi, 1997) translations and English-language adaptations for use in Australia/New Zealand, Canada, and the UK. For information about the availability of SF-36 translations, go to the Internet at http://www.SF-36.com.

Discussion

McDowell and Newell (1996) attribute the "meteoric rise to prominence" observed for the SF-36 Health Survey to a variety of factors. The widespread adoption of the SF-36 in general population surveys and clinical trials is evidence that more practical measurement tools are more likely to be used. The standardization of measurement across studies is producing considerable information about norms and benchmarks useful in comparing "well" and "sick" populations and for estimating the burden of specific conditions.

Although many studies appear to be relying on the SF-36 as the principal measure of health outcome, among the most useful studies are those that use it as a "generic core". A generic core battery of measures makes it possible to compare results across studies and populations and accelerates the accumulation of interpretation guidelines that are essential to determining the clinical, economic, and social relevance of differences in health status and outcomes. Because it is short, the SF-36 can be reproduced in a questionnaire with ample room for other more precise general and specific measures. Numerous studies (Wagner, Keller, Kosinski, et al., 1995; Kantz et al., 1992; Nerenz, Repasky, Whitehouse, & Kahkonen, 1992) have adopted this strategy and have illustrated the advantages of supplementing it.

How useful is the SF-36 for purposes of comparing general and specific population groups, relative to longer surveys? Some SF-36 scales have been shown to have 10-20% less precision than the long-form MOS measures that SF-36 scales were constructed to reproduce (McHorney et al., 1992). Ceiling and floor effects, especially for the original Version 1.0, are another noteworthy limitation documented in the literature for some populations. These disadvantages of the SF-36 should be weighed against the fact that many of the alternative long-form measures require 5-10 times greater respondent burden defined in terms of the number of questionnaire items that must be administered. Empirical studies of this tradeoff suggest that the SF-36 provides a practical alternative to longer measures, and that the eight scales and two summary scales rarely miss a noteworthy difference in physical or mental health status in group level comparisons (Ware et al., 1993; Ware et al., 1994; Katz, Larson, Phillips, Fossel, & Liang, 1992). Regardless, the fact that the SF-36 represents a documented compromise in measurement precision (relative to longer MOS and other measures) leading to a reduction in the statistical power of hypothesis testing should be taken into account in planning clinical trials and other studies. To facilitate such planning, tables of the sample sizes required for conventional statistical tests are published in the two SF-36 users" manuals (Ware et al., 1993; Ware et al., 1994). In relation to longer non-MOS measures, such as the Sickness Impact Profile (SIP), the SF-36 has performed equally well or better in detecting differences in health in two studies (Katz et al., 1992; Beaton, Bombardier, & Hogg-Johnson, 1994).

The value of general and specific population norms, which was demonstrated well for the SIP (Bergner, Bobbitt, Carter, & Gilson, 1981) and later for the MOS SF-20 (Stewart, Hays, & Ware, 1988; Stewart, Greenfield, Hays, Wells, Rogers, Berry, McGlynn, & Ware, 1989) and other measures, has also been demonstrated for the SF-36. In addition to the 20 medical conditions described in the MOS and 14 conditions described in the U.S. population norming survey (Ware et al., 1994), other publications have reported descriptive data for patients with cardiac disease (Krousel-Wood & Re, 1994; Jette & Downing, 1994), depressive disorders (Coulehan et al., 1997), epilepsy (Vickrey, Hays, Graber, Rausch, Engel, Brook, 1992; Wagner et al., 1995), diabetes mellitus (Nerenz et al., 1992; Jacobson, de Groot, & Samson, 1994), migraine headache (Osterhaus, Townsend, Gandek, & Ware, 1994) , heart transplant patients (Rector, Ormaza, & Kubo, 1993), ischemic heart disease (Phillips & Lansky, 1992), ischemic stroke (Kappelle, Adams, Heffner, Torner, Gomez, & Biller, 1994), low back pain (Garratt, Ruta, Abdalla, & Russell, 1993; Lansky et al., 1992) , lung disease (Viramontes & O'Brien, 1994), menorrhagia (Garratt et al., 1994), orthopedic conditions leading to knee replacement (Kantz et al., 1992), knee surgery (Katz et al., 1992), and hip replacement (Katz et al., 1992; Lansky et al., 1992), and for renal disease (Kurtin, Davies, Meyer, DeGiacomo, & Kantz, 1992; Meyer, Espindle, DeGiacomo, Jenuleson, Kurtin, & Davies, 1994; Benedetti, Matas, Hakim, Fasola, Gillingham, McHugh, & Najarian, 1994). Whereas some of the initial descriptive studies using the SF-36 were performed primarily to validate scale scores (McHorney et al., 1992), on the strength of validation studies to date, SF-36 scales appear to be increasingly accepted as valid health measures for purposes of documenting disease burden. Much remains to be discovered about population health in comprehensive terms of functional health and well-being, the relative burden of disease, or the relative benefits of alternative treatments. One reason has been the lack of practical measurement tools appropriate for widespread use across diverse populations. The SF-36 was constructed to provide a basis for such comparisons of results.

As predicted when it was first published (Ware & Sherbourne, 1992), the SF-36 has been widely adopted because of its brevity and its comprehensiveness. Although these two measurement goals are competing, the SF-36 appears to have achieved a psychometrically-sound compromise between them. Population and large-group descriptive studies and clinical trials to date demonstrate that the SF-36 is very useful for descriptive purposes such as documenting differences between sick and well patients and for estimating the relative burden of different medical conditions. Although its usefulness in capturing differences in health outcomes in clinical trials was doubted by many, experience to date from nearly 400 randomized controlled clinical trials suggests that the SF-36 is also a useful tool for evaluating the benefits of alternative treatments (Turner-Bowker et al., 2002).

Although the foundation grants that made the SF-36 Health Survey possible and that subsidized its distribution ended long ago, demand for permissions to use the SF-36 in academic research and in commercial applications in health care have increased markedly in recent years. In response, the Medical Outcomes Trust (MOT), Health Assessment Lab (HAL), and QualityMetric Incorporated - the organizations holding all SF-36 copyrights and trademarks - have established common policies for granting permissions for use of the original and improved forms and all translations. In January, 2002 these three organizations merged their licensing programs for both scholarly research and commercial applications and they offered simplified online processing services. All licensing services are now explained and are available on the Internet at: http://www.sf-36.com and http://www.qualitymetric.com. As discussed in greater detail on the two websites above and on the MOT website, the goals of these three organizations include: (a) maintaining the scientific standards for surveys and scoring algorithms that make results directly comparable and interpretable; (b) making surveys available royalty free to individuals and organizations who collect their own data for purposes of scholarly research; and, (c) a commercial licensing program that includes royalty payments by those who profit from the use of the intellectual property in support of the research community that is advancing the state of the art. The response to the merged and simplified licensing program has been very favorable from both the scientific community and industry as evidenced by the more than one thousand licenses that have been granted to academic researchers, pharmaceutical companies, data collection vendors, health care providers, government agencies and others in 2002.
